# *Clostridium difficile* Enteritis With NAP7/078 Toxigenic Strain

**DOI:** 10.1093/ofid/ofad523

**Published:** 2023-10-26

**Authors:** Rahel T Zewude, Sami A Chadi, Eve Capistran, Isaac I Bogoch

**Affiliations:** Division of Infectious Diseases, Department of Medicine, University of Toronto, Toronto, Canada; Division of General Surgery and Surgical Oncology, Department of Surgery, University Health Network, University of Toronto, Toronto, Canada; Department of Microbiology and Infectious Diseases, Faculty of Medicine and Health Sciences, Université de Sherbrooke, Sherbrooke, Canada; Division of Infectious Diseases, Department of Medicine, University of Toronto, Toronto, Canada

To the  Editor—We recently treated a patient with severe *Clostridium difficile* enteritis, and we believe that this rare syndrome would be of interest to *Open Forum Infectious Diseases* readers. We present our case in the context of prior literature and identify salient gaps with the role of toxigenic strains and treatment guidelines for this diagnosis with significant mortality.

Our patient is a 17-year old male with necrotizing enterocolitis (NEC) at birth resulting in intra-abdominal adhesions managed nonoperatively. He had no other comorbidities and was not taking any medications at home. He presented with abdominal pain and vomiting, was diagnosed with small bowel obstruction (SBO), and underwent incomplete lysis of congenital or possibly post-NEC adhesions. The patient received preoperative intravenous cefazolin (2-g dose) but no other antibiotics over the preceding 3 months. Two weeks later, he was diagnosed with recurrent SBO with a distended small bowel on computed tomography. A stool *C difficile* toxin test returned positive (Aries polymerase chain reaction for toxin A and B detection). He was given intravenous metronidazole (500 mg every 8 hours) and enteral vancomycin (125 mg every 6 hours). He became hemodynamically unstable and underwent an urgent laparotomy, including resection of 1.5 m of ileum with intraoperative findings of pseudomembranous enteritis. He received a double-barrel stoma with ileomucocutaneous fistula via a Foley catheter. Vancomycin (500 mg every 6 hours) was administered through this catheter antegrade into the colon and retrograde into the proximal ileum, as well as antegrade through a nasogastric tube and retrograde through a rectal tube.

Small intestine tissue grew *C difficile* on culture. Molecular genotyping returned positive for the NAP7/078 toxigenic strain (North American pulsed-field gel electrophoresis type 7) and negative for NAP1/027. He completed a 4-week course of antibiotics and was discharged home. Following clinical and radiographic improvement, he underwent reversal of stoma 5 months later. A week after stoma reversal, he developed watery diarrhea and fever without hemodynamic instability. A stool toxin test returned positive for *C difficile* with genotyping redemonstrating the NAP7/078 strain. Computed tomography showed diffuse distension of small and large bowel without toxic megacolon. He received 10 days of fidaxomicin (200 mg every 12 hours) with symptom resolution.

Freiler et al described 10 cases of *C difficile* enteritis in their 2001 publication. The article identified prior abdominal surgery as an associated comorbidity that was present in 90% of cases, as well as inflammatory bowel disease, which was present in 50% of cases [1]. However, no data regarding specific strains were reported. Klimko et al provided an updated review of *C difficile* enteritis in 2022, describing 77 cases over the last 20 years, also without data on toxigenic strains [2]. Interestingly, in one of the publications included in this review, Lavallée et al described the first case of *C difficile* enteritis caused by the NAP1/027 strain and hypothesized that NAP1/027 may be able to colonize small bowel more easily [3, 4]. They suspected that several of their 12 fatal cases of *C difficile* enteritis may have resulted from this toxigenic strain given its high prevalence at their hospitals with NAP1/027 nosocomial epidemics in preceding years [4, 5]. The NAP7/078 strain, in contrast, has been associated with community onset and younger patients but similar severity when compared with the NAP1/027 strain [6, 7]. There have been no prior reports of NAP7/078 *C difficile* enteritis.

The 2021 Infectious Diseases Society of America update on *C difficile* management does not provide recommendations for *C difficile* enteritis [8]. In the reviews from Freiler et al [1] and Klimko et al [2], dual therapy with parenteral metronidazole and enteral vancomycin was used in most cases. Klimko et al also described vancomycin enemas per distal limb of conduit in several patients with stomas.

Given the hemodynamic instability that persisted in our patient for a few more days beyond ileal resection and the lack of guidance around the management of severe enteritis, we administered vancomycin with 4 routes in conjunction with parenteral metronidazole. Our patient also did not have the commonly described associated comorbidities, such as prior abdominal surgery or inflammatory bowel disease. We suspect that his presentation was likely compounded by an inability to evacuate the *C difficile* toxin from a toxigenic strain through gastroenterocolic routes. The patient likely had a chronically obstructed state with NEC at birth with intra-abdominal adhesions that led to his SBO, which was incompletely addressed, leading to a further obstructive process.

Although a rare and perhaps underdiagnosed entity, *C difficile* enteritis has been increasingly reported over the last 2 decades, and it carries a mortality rate of up to 30% [2, 9]. There is a knowledge gap in the role of toxigenic strains in *C difficile* enteritis, and genotyping in future cases can further our understanding of this.

## Notes

***Author contributions.*** R. T. Z. conceptualized the study and contributed to data curation, writing of the first draft of the article, and reviewing. S. A. C. and I. I. B. contributed to data curation, writing of the first draft of the article, and reviewing. E. C. contributed to data curation and review of the article. S. A. C. and I. I. B. supervised the study as senior authors. All authors reviewed and revised the manuscript and approved its final version to be submitted for publication. All authors were involved in the patient's clinical care.

***Patient consent statement.*** The patient's verbal consent was obtained. Note that the case presented in this letter to the editor does not include any patient identifiers or images.
